# Young pregnant woman with double orifice mitral valve presented with severe mitral regurgitation

**DOI:** 10.1093/ehjcr/ytae403

**Published:** 2024-08-06

**Authors:** Vasileios Bouratzis, Lampros Lakkas, Nikolaos Zois, Nikoleta Douskou, Katerina K Naka

**Affiliations:** Second Department of Cardiology, University Hospital of Ioannina, Stavros Niarchos Avenue, Ioannina 45 500, Greece; Second Department of Cardiology, University Hospital of Ioannina, Stavros Niarchos Avenue, Ioannina 45 500, Greece; Second Department of Cardiology, University Hospital of Ioannina, Stavros Niarchos Avenue, Ioannina 45 500, Greece; Second Department of Cardiology, University Hospital of Ioannina, Stavros Niarchos Avenue, Ioannina 45 500, Greece; Second Department of Cardiology, University Hospital of Ioannina, Stavros Niarchos Avenue, Ioannina 45 500, Greece

A 24-year-old pregnant female (24 weeks) was referred to the outpatient clinic for evaluation. She had a history of surgically corrected partial atrioventricular canal defect (AVCD) (17 years ago) with only residual left to right shunt, moderate mitral regurgitation (MR) due to double orifice mitral valve (DOMV) with a cleft due to AVCD, and bicuspid aortic valve, and 2 years ago, she underwent transoesophageal echo (TOE) with 3D reconstruction imaging (*Panel A1–A4*; Supplementary material online, *Videos S1*–*S3*) and cardiac magnetic resonance (CMR) (*Panel B1 and B2*) which confirmed DOMV causing moderate MR.

**Figure:**
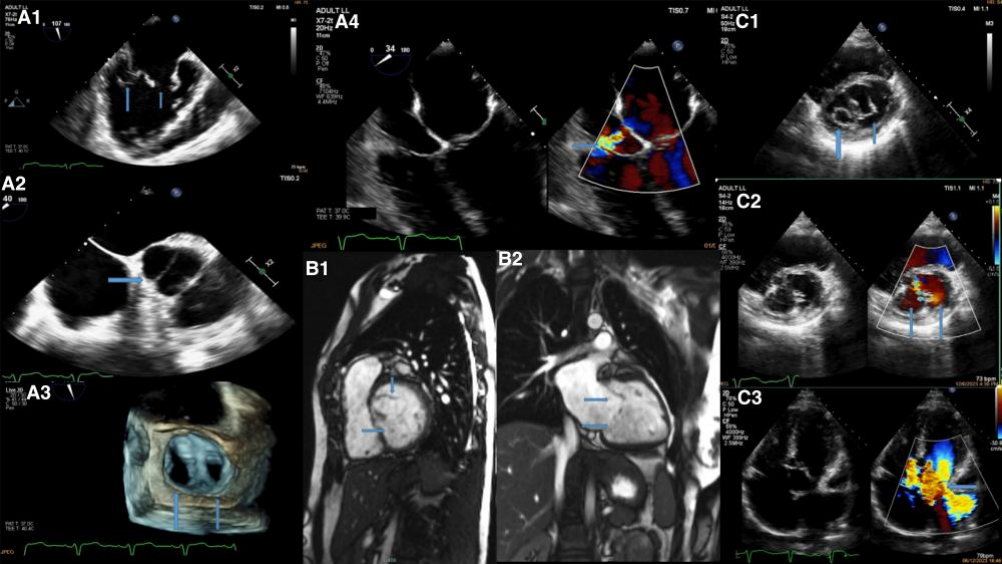


A transthoracic echocardiogram showed preserved left ventricular systolic function, biatrial dilation, bicuspid aortic valve with moderate regurgitation, DOMV with severe MR due to hyperdynamic conditions (pregnancy), and a dilated right ventricle with normal systolic function (*Panel C1–C3*; Supplementary material online, *Video S4*).

Double orifice mitral valve is a very rare cardiac congenital anomaly (incidence 1 in 100 000) of the mitral valve apparatus consisting of an accessory bridge of fibrous tissue, which partially or completely divides MV into two orifices, usually being a stenotic condition. It is often associated with other congenital diseases such as atrioventricular septal defects, patent ductus arteriosus, and coarctation of aorta. Even though TOE is the gold standard method for the diagnosis, CMR can also be used in challenging cases. Here, we present a rare case of a young pregnant woman with DOMV presented with severe MR due to hyperdynamic circulation that is a common situation that occurs in pregnancy.

(*Panel A1*) Transoesophageal echo mid-oesophageal bicommisural view showing double orifice mitral valve. (*Panel A2*) Transoesophageal echo mid-oesophageal short-axis view showing bicuspid aortic valve. (*Panel A3*) Transoesophageal echo with 3D reconstruction (surgeon’s view) showing double orifice mitral valve. (*Panel A4*) Transoesophageal echo mid-oesophageal four-chamber view showing surgically corrected partial atrioventricular canal defect with residual left to right shunt. (*Panel B1*) Cardiac magnetic resonance short-axis view showing double orifice mitral valve. (*Panel B2*) Cardiac magnetic resonance apical long-axis view showing double orifice mitral valve with mitral regurgitation. (*Panel C1*) Transthoracic echocardiogram short-axis view showing double orifice mitral valve. (*Panel C2*) Tranthoracic echocardiogram short-axis colour Doppler view showing double orifice mitral valve. (*Panel C3*) Transthoracic echocardiogram four-chamber view with colour Doppler showing double orifice mitral valve with severe mitral regurgitation.

## Supplementary Material

ytae403_Supplementary_Data

## Data Availability

The data underlying this article are available in the article and in its online Supplementary material.

## Notes

**Consent:** The authors confirm that written consent for submission and publication of this case report including images and associated text has been obtained from the patient in line with the Committee on Publication Ethics guidelines.

**Funding:** None declared

